# Access to Care and Use of the Internet to Search for Health Information: Results From the US National Health Interview Survey

**DOI:** 10.2196/jmir.4126

**Published:** 2015-04-29

**Authors:** Daniel J Amante, Timothy P Hogan, Sherry L Pagoto, Thomas M English, Kate L Lapane

**Affiliations:** ^1^Division of Health Informatics and Implementation ScienceDepartment of Quantitative Health SciencesUniversity of Massachusetts Medical SchoolWorcester, MAUnited States; ^2^Center for Healthcare Organization and Implementation Research (CHOIR)eHealth Quality Enhancement Research Initiative (QUERI)National eHealth QUERI Coordinating CenterBedford, MAUnited States; ^3^Division of Preventive and Behavioral MedicineDepartment of MedicineUniversity of Massachusetts Medical SchoolWorcester, MAUnited States; ^4^Division of Epidemiology of Chronic Diseases and Vulnerable PopulationsDepartment of Quantitative Health SciencesUniversity of Massachusetts Medical SchoolWorcester, MAUnited States

**Keywords:** health services accessibility, health information technology, information-seeking behavior, Patient Protection and Affordable Care Act

## Abstract

**Background:**

The insurance mandate of the Affordable Care Act has increased the number of people with health coverage in the United States. There is speculation that this increase in the number of insured could make accessing health care services more difficult. Those who are unable to access care in a timely manner may use the Internet to search for information needed to answer their health questions.

**Objective:**

The aim was to determine whether difficulty accessing health care services for reasons unrelated to insurance coverage is associated with increased use of the Internet to obtain health information.

**Methods:**

Survey data from 32,139 adults in the 2011 National Health Interview Study (NHIS) were used in this study. The exposure for this analysis was reporting difficulty accessing health care services or delaying getting care for a reason unrelated to insurance status. To define this exposure, we examined 8 questions that asked whether different access problems occurred during the previous 12 months. The outcome for this analysis, health information technology (HIT) use, was captured by examining 2 questions that asked survey respondents if they used an online health chat room or searched the Internet to obtain health information in the previous 12 months. Several multinomial logistic regressions estimating the odds of using HIT for each reported access difficulty were conducted to accomplish the study objective.

**Results:**

Of a survey population of 32,139 adults, more than 15.90% (n=5109) reported experiencing at least one access to care barrier, whereas 3.63% (1168/32,139) reported using online health chat rooms and 43.55% (13,997/32,139) reported searching the Internet for health information. Adults who reported difficulty accessing health care services for reasons unrelated to their health insurance coverage had greater odds of using the Internet to obtain health information. Those who reported delaying getting care because they could not get an appointment soon enough (OR 2.2, 95% CI 1.9-2.5), were told the doctor would not accept them as a new patient or accept their insurance (OR 2.1, 95% CI 1.7-2.5 and OR 2.1, 95% CI 1.7-2.5, respectively), or because the doctor’s office was not open when they could go (OR 2.2, 95% CI 1.9-2.7) had more than twice the odds of using the Internet to obtain health information compared to those who did not report such access difficulties.

**Conclusions:**

People experiencing trouble accessing health care services for reasons unrelated to their insurance status are more likely to report using the Internet to obtain health information. Improving the accuracy and reliability of health information resources that are publicly available online could help those who are searching for information due to trouble accessing health care services.

## Introduction

### Background

The passing of the Patient Protection and Affordable Care Act (ACA) of 2010 will significantly increase the number of people insured in the United States by expanding Medicaid, subsidizing insurance for lower-income Americans, and mandating that everyone has coverage or else be forced to pay an additional tax [1]. Although the insurance mandate of the ACA addresses a major barrier to accessing health care services, it may also introduce unintended consequences. *Access to care* is defined as the opportunity to reach and obtain appropriate health care services to satisfy a perceived need [2]. Although access to care will improve for the newly insured, the concept of moral hazard suggests that this increase in insurance will greatly affect the allocation of resources because those who gain coverage will use medical care that they otherwise would not have used if they were required to pay its full cost [3,4]. Taking this predicted increase of health care service utilization into consideration along with the documented shortage of primary care physicians [5] and increase in disease prevalence for the baby boomer generation [6], quickly accessing health care services when needed could become much more difficult in the near future [7]. Despite these substantial implications, little is known about how people who have insurance but are unable to access care in a timely manner are obtaining information to answer their health questions.

The proliferation of the Internet has provided an opportunity for patients to search for and gather medical information, albeit of unknown accuracy and reliability, that was previously unavailable to them. An estimated 74% of adults in the United States go online to access the Internet with up to 80% of them looking for health information online [8]. The Internet can improve health outcomes by increasing the availability of information, providing social support, improving feelings of self-efficacy, and facilitating interaction with the health care system [9,10]. Online health information seeking may also serve as an alternative to more traditional methods of obtaining health information, such as receiving information directly from health care providers, particularly for those who have trouble immediately accessing health care services when needed. Cline and Haynes [11] described 3 ways consumers access online health information: searching directly for health information, participating in support groups, and consulting with health professionals. Patients with access to providers will inevitably use the Internet more to perform functions that involve interaction with their clinical team than those without access to providers. These tasks could include using a health care organization’s Web-based portal to schedule an appointment online, to communicate with a provider over secure messaging, to request prescription refills, or to participate in a telehealth consultation. However, no portal or contact with a health care provider is needed to search the Internet for health information or to use online support groups or health chat rooms to learn more about health topics. Patients who are unable to obtain health care services quickly may turn to such publically available resources on the Internet to address their immediate needs.

### Objective

We looked to investigate the relationship between health care access barriers unrelated to insurance coverage and the use of the Internet to retrieve health information. We hypothesized that those who report trouble accessing services will be more likely to search the Internet for health information. To test this hypothesis, we analyzed data from a large, nationally representative sample of noninstitutionalized adults living in the United States. Determining whether patients use the Internet to satisfy a need they are unable to address with traditional health care services could lead to an opportunity to deliver accurate, reliable, and tailored online health resources to certain people. Improving the quality of resources available on the Internet and disseminating them to the appropriate audience could help improve health outcomes, ease frustration due to insufficient access to health care, and reduce expensive use of emergency services.

## Methods

### Dataset

This study used data from the 2011 National Health Interview Survey (NHIS). The NHIS is a multipurpose health survey conducted by the National Center for Health Statistics (NCHS) and the Centers for Disease Control and Prevention (CDC) annually since 1957 [12]. The NHIS is the principal source of information on the health of the civilian noninstitutionalized household population of the United States. Data are collected through personal household interviews by census interviewers using computer-assisted personal interviewing (CAPI). The NHIS is made up of a core questionnaire that is periodically revised but remains mostly stable year to year. It also contains a supplement questionnaire containing questions on current health topics that vary every year. The version we used (2011) contained supplement questions related to health information technology (HIT) use.

### Sample

For each family, 1 child aged 17 years or younger and 1 adult aged 18 years or older were randomly selected to complete the Sample Child and Sample Adult questionnaires. These questionnaires differ slightly and produce 2 different data files. We used the Sample Adult data file for this study. The 2011 NHIS collected data from 33,014 adults aged 18 years or older, with 465 individuals having a proxy answer the questions due to being physically or mentally unable to answer themselves. Individuals who had a proxy answer or who had missing data on any of the variables of interest were excluded from this analysis. The response rate for the Sample Adult survey was 81.60% (33,014/40,458) of eligible adults. The NHIS has oversampled the black population since 1985, the Hispanic population since 1995, and the Asian population since 2006. Additionally, the 2011 NHIS selection process was revised so that black, Hispanic, or Asian individuals who were older than 65 years had an increased chance of being selected as the sample adult for their household. Greater details about the NHIS sampling methodology can be found elsewhere [12].

### Measures

#### Operational Definition of Exposure-Health Service Access Difficulty

The exposure for this analysis was having difficulty accessing health care services or delay getting care for a reason unrelated to insurance status. To define this exposure, we looked at several different questions included in the NHIS. First, we looked at 8 questions asking whether any specific access problems occurred during the past 12 months. These questions were:

1. Did you have any trouble finding a general doctor or provider who would see you?
2. Were you told by a doctor’s office or clinic that they would not accept you as a new patient?
3. Were you told by a doctor’s office or clinic that they did not accept your health care coverage?
4. Have you delayed getting care because you could not get through on the telephone?
5. Have you delayed getting care because you could not get an appointment soon enough?
6. Have you delayed getting care because once you get there you have to wait too long to see the doctor?
7. Have you delayed getting care because the clinic/doctor’s office was not open when you could get there?
8. Have you delayed getting care because you did not have transportation?

All 8 questions had the response options of yes, no, refused, not ascertained, and don’t know. Those who refused to answer, an answer was not ascertained, or answered “don’t know” on any of the questions (n=338) were excluded from the analysis after determining no significant differences existed between them and those to be included in the analysis.

#### Operational Definition of Outcome-Health Information Technology Use

The outcome of interest was using the Internet to obtain health information. To define this outcome, we considered 2 questions in the HIT supplement that focused on activities conducted in the preceding 12 months. These questions asked specifically about the use of computers to access the Internet and did not ask about the use of mobile phones and/or tablets. The questions were:

1. During the past 12 months, have you ever used computers to look up health information on the Internet?
2. During the past 12 months, have you ever used computers to use online chat groups to learn about health topics?

From these 2 questions, we categorized 2 mutually exclusive categories of HIT use for the outcome variable. The first category (“HIT use”) consisted of those who reported using online chat groups to learn about health topics and/or those who reported using the Internet to search for health information. The second group (“no HIT use”) contained all who reported they did not use computers to search for health information on the Internet or online chat rooms to learn about health topics in the previous 12 months.

#### Additional Covariates

Use of HIT has been found to vary by age [13,14], sex [13,15], race/ethnicity [16], education level [13,14], and marital status [17]. To account for such differences, data were collected on these demographic and socioeconomic covariates. We categorized age into 3 groups (18-35 years, 35-60 years, and ≥60 years), race/ethnicity into 5 groups (non-Hispanic white, Hispanic, Asian, non-Hispanic black, and other/multiple races), education into 4 groups (less than high school, high school diploma/GED, Bachelor’s/Associate’s degree, and advanced degree), and marital status into 4 groups (married/live with partner, widowed, divorced/separated, and never married). Due to missing household income data, we considered education level as a proxy variable. This allowed for every case in the analysis to have complete data on all variables. People who are sick or have a chronic disease are also more likely to utilize HIT resources [14,18]. To adjust for health status, we looked at 3 variables in the model: self-reported health status (5-point scale from 1=poor to 5=excellent), limitation due to chronic disease (yes/no), and the Charlson Comorbidity Index (CCI) [19]. The CCI values ranged from 0-17 based on the presence of the following medical conditions: myocardial infarction, cerebrovascular disease, chronic pulmonary disease, ulcer disease, cancer, diabetes, renal disease, liver disease, connective tissue disease, and dementia as described in a previous study using NHIS data [20]. A score of zero indicates no comorbidities with higher scores representing a higher predicted risk of mortality attributable to comorbidities [21]. We categorized the CCI scores into 3 groups containing adults with a CCI of 0, 1, and ≥2. Insurance coverage was categorized as having any private insurance coverage, public insurance coverage, or having no insurance coverage.

### Analytic Methods

First, weighted percentages within each category of sociodemographic and health status variables separated by HIT use were calculated. Differences within each variable were examined using the Pearson chi-square test for categorical variables. Next, a multinomial logistic regression including all sociodemographic and health status variables was used to calculate adjusted odds ratios (AORs) describing the odds of using HIT compared to the odds of not using HIT within each variable. To accomplish the study objective, multiple multinomial logistic regressions were conducted looking at differences in the odds of using HIT compared to the odds of not using HIT to obtain health information. The first multinomial regression we ran for each exposure variable included only the outcome of interest (HIT use), producing crude ORs for each outcome variable. We then added sociodemographic and health status covariates to each model to account for sex, age, race, education, marital status, health status, and insurance coverage, producing AORs. We chose these covariates because the literature has shown that they significantly differ in respect to HIT use. To account for oversampling, a known nonzero probability of selection for each individual was used in conjunction with adjustments for nonresponse and poststratification to generate sample weights for each individual that were applied to the regressions [12].

## Results

### Sample Population Characteristics

Of the 33,014 adults surveyed in the 2011 NHIS, 32,139 (97.35%) were included in this analysis. Of those included, 3.63% (1168/32,139) used online health chat rooms to learn about health topics, 43.55% (13,997/32,139) used the Internet to search for health information, and 56.19% (18,059/32,139) did not search the Internet or use online chat rooms to find health information. The majority (92.89%, 1085/1168) of those who used online health chat rooms also reported using the Internet to search for health information in the previous 12 months. Distribution of sociodemographic and health status variables within each category of HIT use are presented in Table 1. All differences were statistically significant (*P*<.001) as determined by the Pearson chi-square test.

Younger participants were more likely to use the Internet to search for health information; 52.84% of participants aged 18-35 years and 51.80% aged 35-60 years reported using either online health chat rooms or the Internet to search for health information compared to only 31.35% of adults aged 60 years or older (data not shown). Women had more than 1.5 times greater odds than men to report using the Internet to search for health information (OR 1.8, 95% CI 1.7-1.9). White participants reported using the Internet to search for health information most frequently (50.01%, 9549/19,095) compared to other races/ethnicities (Hispanic: 30.52%, 1663/5448; Asian: 45.98%, 910/1979; black: 33.44%, 1580/4725; other/multiple races: 42.38%, 378/892). The percentage of adults using HIT to search for health information increased with education level (13.16%, 698/5304 with <high school; 38.94%, 5700/14,637 with high school diploma/GED; 60.47%, 5469/9044 with Bachelor’s/Associate’s degree; and 70.16%, 2213/3154 with advanced degree). Adults who were widowed used the Internet to search for health information less than those who were married, had never married, or were divorced/separated (18.50%, 549/2968 compared to 48.45%, 7822/16,145; 40.08%, 2201/5492; and 46.56%, 3508/7534; respectively). The odds ratios of using HIT compared to not using HIT for sample characteristics are presented in Table 1.

Reported use of HIT to obtain health information did not change as self-reported health status worsened. Those who reported a limitation due to a chronic condition used the Internet to search for health information less than those who reported no limitation due to a chronic condition (Table 1). Adults with a CCI of zero had lower odds of reporting use of either HIT tool to find health information compared to those with a CCI of 1 (OR 1.3, 95% CI 1.2-1.4) and those with a CCI of 2 or greater (OR 1.4, 95% CI 1.3-1.5). Adults who reported having private insurance coverage had approximately 1.5 times greater odds of using the Internet to search for health information compared to those who were not covered (OR 1.5, 95% CI 1.4-1.6) (Table 1).

**Table 1:** Sample characteristics by health information technology (HIT) use among adults, NHIS 2011.

Variable		HIT use n=14,080		No HIT use (ref) n=18,059
		%^a^	OR (95% CI)^b^	%^a^
**Age (years)**				
	18-34	34.72	1.0 (ref)	27.48
	35-60	48.57	0.7 (0.6-0.7)	40.08
	≥60	16.71	0.4 (0.3-0.4)	32.44
**Sex**				
	Men	42.80	1.0 (ref)	53.47
	Women	57.20	1.8 (1.7-1.9)	46.53
**Race/ethnicity**				
	Non-Hispanic white	74.81	1.0 (ref)	61.91
	Hispanic	9.37	0.6 (0.5-0.6)	17.16
	Asian	4.62	0.6 (0.5-0.6)	4.79
	Non-Hispanic black	8.83	0.6 (0.5-0.6)	13.67
	Other/multiple races	2.47	0.9 (0.8-1.1)	2.47
**Education**				
	<High school	4.98	1.0 (ref)	22.48
	High school diploma/GED	41.61	2.8 (2.5-3.2)	51.09
	Bachelor’s/Associate’s	38.00	5.7 (5.0-6.4)	21.01
	Advanced degree	15.41	9.8 (8.2-11.2)	5.42
**Marital status**				
	Married/live with partner	66.20	1.0 (ref)	56.51
	Widowed	2.62	0.5 (0.4-0.5)	8.84
	Divorced/separated	10.35	0.8 (0.8-0.9)	12.67
	Never married	20.83	0.8 (0.7-0.9)	21.98
**Self-reported health status**				
	Excellent/very good	67.29	1.0 (ref)	54.65
	Good	23.57	1.0 (0.9-1.0)	28.59
	Fair/poor	9.13	0.8 (0.8-0.9)	16.75
**Limited due to chronic condition**				
	No	88.56	1.0 (ref)	80.32
	Yes	11.44	1.0 (0.9-1.1)	19.68
**Charlson Comorbidity Index**				
	0	66.19	1.0 (ref)	60.28
	1	18.75	1.3 (1.2-1.4)	19.21
	≥2	15.06	1.4 (1.3-1.5)	20.50
**Insurance status**				
	Not covered	13.64	1.0 (ref)	20.17
	Private coverage	66.87	1.5 (1.4-1.6)	41.46
	Public coverage	19.49	0.9 (0.8-1.0)	38.37

^a^ All differences in weighted percentages statistically significant (*P*<.001) as determined by the Pearson chi-square test.

^b^ All ORs are adjusted odds of using HIT compared to odds of no HIT use (reference group) accounting for all variables.

### Health Care Access Difficulties

**Figure 1:**
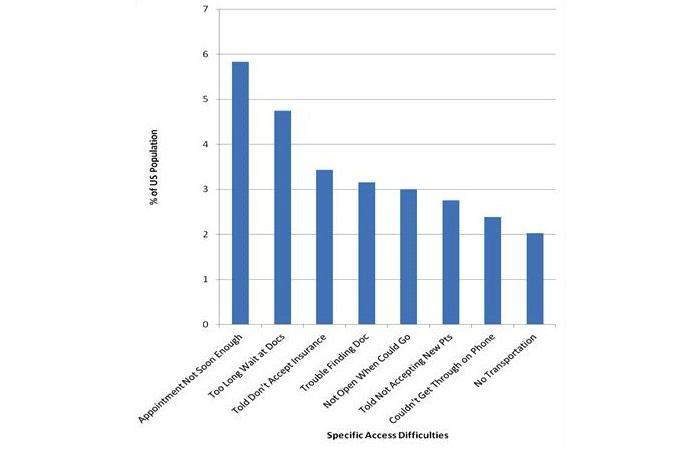
Percent of US population reporting each access difficulty over the past 12 months.

Of the 5109 adults who reported experiencing any of the access difficulties in the previous 12 months, 55.47% (2834/5109) reported having experienced only 1 of the 8 specific access difficulties, 22.29% (1139/5109) reported experiencing 2 of the specific access difficulties, 12.92% (660/5109) reported experiencing 3 specific access difficulties, and 9.31% (476/5109) reported experiencing 4 or more of the specific access difficulties in the past 12 months (data not shown). The most commonly reported difficulties accessing health care services in the past 12 months were delaying getting care because they could not get an appointment soon enough (6.09%, 1956/32,139) and delaying getting care because once they got there, they had to wait too long to see the doctor (5.23%, 1681/32,139). The percentage of adults in the US population who reported experiencing each specific type of difficulty are listed in Figure 1.

### Relationship Between Health Care Access Difficulty and Health Information Technology Use

The results in Table 2 show the crude and adjusted odds of using HIT to obtain health information for each of the access difficulty questions. Those who reported delays in care because they were told the doctor would not accept them as a new patient or accept their insurance, because they could not get an appointment soon enough, or because the doctor’s office was not open when they could go had more than twice (OR 2.1, 95% CI 1.7-2.5; OR 2.1, 95% CI 1.7-2.5; OR 2.2, 95% CI 1.9-2.7; OR 2.2, 95% CI 1.9-2.7; respectively) the odds of reporting use of Internet tools to search for health information after accounting for covariates. Those who reported having trouble finding a doctor who would see them or delayed getting care because they could not get through on the phone or because the wait was too long had more than 1.5 times the odds of reporting use of HIT to search for health information (OR 1.8, 95% CI 1.5-2.1; OR 1.9, 95% CI 1.6-2.4; OR 1.5, 95% CI 1.4-1.8; respectively) after accounting for covariates. Lastly, those who reported delaying care because they did not have transportation were approximately 1.4 times more likely to report use of HIT (OR 1.4, 95% CI 1.1-1.7) after accounting for covariates.

**Table 2:** Relationship between specific access difficulties over the past 12 months and health information technology (HIT) use.

Specific access difficulty	HIT use n=14,080			No HIT use (ref) n=18,059
	%^a^	OR (95% CI)^b^	AOR (95% CI)^c^	%^a^
Trouble finding a doctor who would see you	3.67	1.38 (1.18-1.60)	1.80 (1.53-2.13)	2.70
Told doctor would not accept you as new patient	3.53	1.73 (1.45-2.06)	2.07 (1.70-2.51)	2.07
Told doctor would not accept your insurance	4.49	1.84 (1.58-2.15)	2.05 (1.71-2.45)	2.49
Delayed getting care because could not get through on phone	3.05	1.71 (1.43-2.04)	1.93 (1.58-2.36)	1.81
Delayed getting care because could not get an appointment soon enough	7.97	2.12 (1.89-2.38)	2.21 (1.94-2.51)	3.93
Delayed getting care because once there, wait was too long to see doctor	5.23	1.22 (1.09-1.38)	1.54 (1.35-1.77)	4.31
Delayed getting care because doctor’s office not open when you could go	4.25	2.30 (1.96-2.69)	2.23 (1.88-2.65)	1.90
Delayed getting care because did not have transportation	1.78	0.78 (0.66-0.93)	1.39 (1.14-1.69)	2.25

^a^ Weighted percentage.

^b^ Crude ORs are odds of using HIT compared to odds of no HIT use (reference group).

^c^ Adjusted ORs accounting for sex, age, race, education, marital status, self-reported health status, presence of chronic disease, CCI, and insurance coverage.

## Discussion

This analysis of a large, nationally representative population of adults revealed that more than 15% of US adults reported experiencing difficulty accessing health care services over the previous 12 months for a variety of reasons. The most common difficulties reported were patients delaying getting care because they either could not get an appointment soon enough or because the wait at the doctor’s office was too long. We also found that more than 40% of adults use the Internet to search for health information and approximately 3.7% of adults use online health chat rooms. These results are consistent with previously published studies [8,22].

Who is using the Internet to search for health information and for what purposes needs to be better understood so that online resources can be tailored and improved to maximize its usefulness. Negative consequences of medical Internet use, however, include delivery of inaccurate information, loss of private health information, and potential for harm due to inappropriate or misleading information [23]. Although it has been previously reported that those with higher income [14,22], greater education [13,14,22], of female gender [13-15], younger in age [13,14,24], of nonminority status [16], married [17], and with chronic [14,18] or stigmatized diseases [25] are more likely to use the Internet for health purposes, many of these differences may dissipate after accounting for recent changes in Internet access [15].

As access to the Internet continues to increase across all groups of people, it is important to monitor which patients are turning to the Internet to search for health information, why and how they are using it, and what the resulting effects on health outcomes are. To our knowledge, this study is the first to report that people who experience trouble obtaining needed health services for reasons unrelated to insurance status use the Internet to search for health information more than those who do not have issues accessing services. These findings may have implications for the decreased availability of health care services due to increased insurance coverage resulting from the insurance mandate of the ACA.

Although studies have shown that extending hours of primary care offices reduce the number of emergency room visits [26], a 2007 survey showed that only 28% of adults reported that their regular practice offered hours outside the normal workday or on the weekends, a percentage far less than other industrialized nations [27]. Furthermore, many have expressed concerns that the ACA will further exacerbate the primary care shortage dilemma as citizens who gain public coverage or are mandated to have health coverage begin accessing services at a greater frequency [28], as happened to Massachusetts in 2006 after passing a similar insurance mandated health reform law [29]. As the delays of obtaining primary care appointments continue to increase, the potential for the Internet to help those in need of health information increases as well. For the Internet to be truly helpful, however, people need to know where to find accurate and reliable information, and how to utilize such information to improve their health conditions.

People with a CCI of 1 or more had greater odds of using the Internet for online health chat rooms or to search for health information than those with a CCI of zero. This makes sense because those with comorbidities may rely on the Internet to obtain information about their illnesses. However, people who rated their health as fair/poor had lower odds of searching the Internet for health information compared to those who reported excellent/very good health status. Similarly, those who reported having functional limitation due to a chronic disease also had lower odds of searching the Internet for health information than those who reported no limitation due to a chronic disease. Although previous reports suggest that sicker participants use HIT tools more often [14,18], including HIT tools provided by their care teams such as online prescription refills, secure messaging, and appointment requests, our analysis was only focused on the publically available HIT tools of searching for health information or online health chat rooms. This limited sample of HIT focus may explain some of the inconsistencies found between our results and the literature, as those who reported poorer health or limitation to chronic conditions could be less likely to search the Internet for health information but more likely to use HIT tools provided by their care team. This study also does not account for Internet access at home. This could explain some of our contradictory findings as previous studies have found that although people with chronic conditions are less likely to have Internet access [30], of those with Internet access, adults with a chronic condition use it more to search for information than those without a chronic condition [18].

We also found that younger people and those with more education have greater odds of reporting use of the Internet to search for health information, consistent with previous studies [13,14,22]. A logical explanation for this is that the health question these adults need answered may not be severe enough, in their opinion, to warrant the full effort or time required to obtain routine, urgent, or emergent clinical attention. Instead of taking time out of their busy day, they may decide to try to resolve the health question on their own. Because this group of adults is more comfortable with the Internet, providing them with easily obtainable, user-friendly, accurate, and reliable online resources could help them make appropriate decisions about how best to maintain or improve their health condition. Additionally, there are providers who offer Web-based video consultations and websites that enable patients to quickly get answers to their health questions from a physician. Although legal and ethical questions regarding compensation, liability, and privacy of personal health information need to be continually addressed [31], this type of innovative delivery of care has potential to improve the accessibility and efficiency of health care services [32]. In addition to increased efficiency and accessibility, a recently published review examined the economic value of clinical telehealth and found that the delivery of home care health services by Web-based video consultations was also cost-effective [33].

This study has several strengths. Because we used data from a nationally representative study, the results produced good estimates of the prevalence of HIT use and the difficulties of accessing health care services in the United States. In addition, our exposure variables (access difficulties) were based on actual examples of problems people have obtaining health care services. This strength is in agreement with the access to care framework suggested by Levesque et al [2], in which access to care is defined as the opportunity to reach and obtain appropriate health care services to satisfy a perceived need. Central to this definition is the process of seeking care. We believe that our exposure questions contain elements of this process and view this agreement as a major strength of our study. The NHIS was also the first nationally representative household survey to collect data on Internet-based HIT use [24]. Furthermore, both the exposure and the outcome variables used the same recall period of 12 months. This increases the meaningfulness of the correlation between the two. Also, due to the wealth of information collected in the survey, we were able to account for several important covariates like race, education, marital status, health status, and insurance coverage. Lastly, since the study population was large and very little data was missing, we were able to include only cases with no data missing from all variables included in the model without needing to exclude a high percentage of adults from the study.

When interpreting the results, it is important to keep the following limitations in mind. The NHIS does not collect data on computer-enabled Internet access at home. Likewise, it also does not monitor Internet access via mobile and tablet devices. Since many low-income and young people may not have access to a computer at home, but may have access to the Internet via mobile phones or tablets, this study may have underestimated HIT use among people who have more difficulty accessing health care services due to financial reasons. We also did not account for differences in HIT use that may occur between patients with not well-understood or stigmatized diseases. Whether the participants were using the Internet to search for health information for themselves or another is an additional limitation. Previous studies have found that almost half of people who go online to search for health information are doing so on behalf of someone else [8]. Lastly, given the large sample population, differences in HIT use should be closely scrutinized to determine if the differences found, while statistically significant, are also meaningful. Since the NHIS is a cross-sectional study, meaningful differences can only be considered as correlational and not causal.

Another important limitation to consider is that the questions we used from the NHIS study only represented a portion of all dimensions that encompass the concept of access to health care. Levesque et al [2] describe 5 different dimensions of access to care: approachability, acceptability, availability and accommodation, affordability, and appropriateness. The majority of the questions we included fall under either the approachability or the availability and accommodation dimensions. No questions in the NHIS contained examples of difficulty accessing services due to dimensions of acceptability, affordability, or appropriateness. Future studies should try to incorporate additional exposure variables that would provide insight into the relationship between difficulty accessing health care services and the use of publically available online health resources.

In conclusion, we found that people who experience difficulty obtaining needed health services use online resources to obtain health information more than those not reporting difficulties obtaining services. With the ACA mandating that all people have health insurance, the patterns of health care utilization will be evolving. If difficulty accessing health care services increase, more patients may turn to resources available to them on the Internet. It needs to be ensured that accurate and reliable resources are available, tailored, and distributed to these people.

## Abbreviations

ACA: Affordable Care Act
CAPI: computer-assisted personal interviewing
CCI: Charlson Comorbidity Index
CDC: Centers for Disease Control and Prevention
HIT: health information technology
NCHS: National Center for Health Statistics
NHIS: National Health Interview Study
